# The Effect of Vorinostat on the Development of Resistance to Doxorubicin in Neuroblastoma

**DOI:** 10.1371/journal.pone.0040816

**Published:** 2012-07-19

**Authors:** Timothy B. Lautz, Chunfa Jie, Sandra Clark, Jessica A. Naiditch, Nadereh Jafari, Yi-Yong Qiu, Xin Zheng, Fei Chu, Mary Beth Madonna

**Affiliations:** 1 Department of Surgery, Lurie Children’s Hospital, Northwestern University, Chicago, Illinois, United States of America; 2 Cancer Biology Program, Children’s Memorial Research Center, Northwestern University, Chicago, Illinois, United States of America; 3 Center for Genetic Medicine, Northwestern University, Chicago, Illinois, United States of America; University of Saarland Medical School, Germany

## Abstract

Histone deacetylase (HDAC) inhibitors, especially vorinostat, are currently under investigation as potential adjuncts in the treatment of neuroblastoma. The effect of vorinostat co-treatment on the development of resistance to other chemotherapeutic agents is unknown. In the present study, we treated two human neuroblastoma cell lines [SK-N-SH and SK-N-Be(2)C] with progressively increasing doses of doxorubicin under two conditions: with and without vorinsotat co-therapy. The resultant doxorubicin-resistant (DoxR) and vorinostat-treated doxorubicin resistant (DoxR-v) cells were equally resistant to doxorubicin despite significantly lower P-glycoprotein expression in the DoxR-v cells. Whole genome analysis was performed using the Ilumina Human HT-12 v4 Expression Beadchip to identify genes with differential expression unique to the DoxR-v cells. We uncovered a number of genes whose differential expression in the DoxR-v cells might contribute to their resistant phenotype, including hypoxia inducible factor-2. Finally, we used Gene Ontology to categorize the biological functions of the differentially expressed genes unique to the DoxR-v cells and found that genes involved in cellular metabolism were especially affected.

## Introduction

Neuroblastoma is the most common extra-cranial solid organ malignancy of childhood. Outcome is heterogeneous and depends on several clinical and biologic factors. Five year survival approaches 90% for infants diagnosed with neuroblastoma in the first year of life, but remains only 65% among all children older than one year-of-age and 30–60% for those with high-risk tumors [1]–[3]. Acquired resistance to commonly used chemotherapeutic agents remains a major barrier to successful therapy, especially among older children with high-risk neuroblastoma. Doxorubicin, along with cisplatin, cyclophosphamide and etoposide, are key components of modern chemotherapy protocols for intermediate and high-risk neuroblastoma [2], [4]. The development of resistance to these commonly used agents can be associated with upregulation of multidrug transporter genes such as *mdr1* and *mrp1*. Upregulation of the genes encoding these drug efflux pumps has been correlated with a poor clinical prognosis [5], [6]. Novel strategies are therefore required to prevent or reverse the development of the multidrug resistant phenotype in order to improve the prognosis for children with high-risk, relapsed and recurrent neuroblastoma.

Vorinostat is a class I and II histone deacetylase (HDAC) inhibitor which has demonstrated efficacy against neuroblastoma in preclinical studies [7]–[9]. Vorinostat was well-tolerated in a phase I study of children with solid organ tumors including neuroblastoma, and demonstrated promising early results [10]. Additional phase I trials of novel protocols incorporating vorinostat are currently ongoing [4]. However, the long-term effect of vorinostat treatment on the expression of genes encoding multidrug efflux pumps and the development of resistance to commonly used chemotherapeutic agents is unknown.

In the current study, we investigated the effect of vorinostat co-treatment on the development of drug resistance using two human neuroblastoma cell lines (SK-N-SH and SK-N-Be(2)C) exposed to progressively increasing concentrations of doxorubicin. Our aims were to determine (1) the functional effect of vorinostat on the long term responsiveness of these cells to doxorubicin, and (2) the genetic changes induced by vorinostat during this process.

## Methods

### Cell Lines, Reagents and Proliferation Assay

Human neuroblastoma (SK-N-SH and SK-N-Be(2) C) lines were purchased from American Type Culture Collection (Rockville, MA). DMEM and fetal bovine serum (FBS) were obtained from Mediatech (Herndon, VA) and Atlanta Biologicals (Atlanta, GA), respectively. Doxorubicin, 3-(4,5-dimethyl-2-thiazolyl)2,5-diphenyl tetrazolium bromide (MTT), and deferoxamine mesylate (DFO) were purchased from Sigma (St. Louis, MO). Vorinostat was purchased from ChemieTek (Indianapolis, IN).

**Figure 1:**
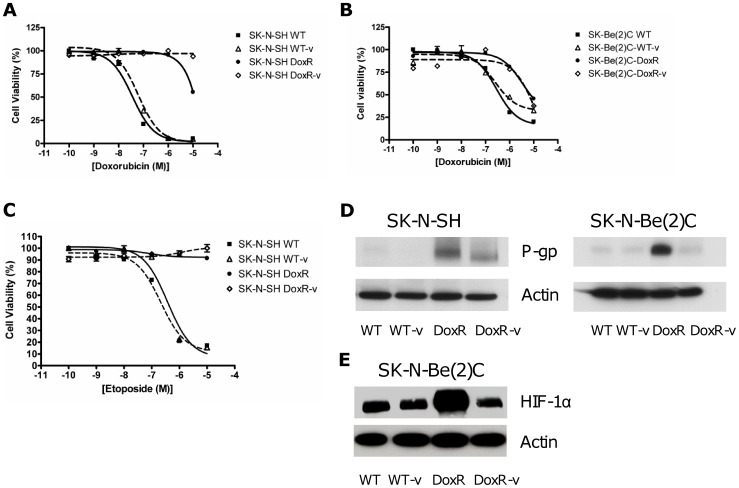
Drug resistance in the SK-N-SH and SK-N-Be(2)C cells. Doxorubicin resistance was assessed by MTT cell proliferation assay in the (a) SK-N-SH and (b) SK-N-Be(2)C cell lines. WT and WT-v proliferation was reduced by increasing concentrations of doxorubicin whilst the DoxR and DoxR-v cells were equally resistant to doxorubicin therapy. (c) Cross resistance to etoposide was likewise confirmed in the SK-N-SH cell line. (d) Western immunoblotting demonstrated significantly greater upregulation of P-glycoprotein (P-gp) in the DoxR than the DoxR-v cells. (e) Likewise in the SK-N-Be(2)C cell line, western blot revealed a pattern of upregulation in hypoxia inducible factor -1α (HIF-1α) similar to that observed with P-gp.

Cell proliferation was assessed by MTT (2-(4,5-Dimethylthiazol-2-yl)-2,5-diphenyltetrazolium bromide) assay. Cells were seeded in 96 well plates and incubated with logarithmic concentrations of doxorubicin ranging from 10^−9^ to 10^−5^ M. MTT (10 µL of 5 mg/ml solution) was added to each well of the titration plate and incubated for 4 hours at 37°C. The cells were then solubilized by the addition of 100 µL of 10% SDS/0.01 mmol/L HCL and incubated for 15 hours at 37°C. The absorbance of each well was determined in an ELISA plated reader using an activation wavelength of 570 nm and a reference wavelength of 650 nm. Cell viability in the presence of different doses of doxorubicin was determined by comparison with untreated control cells.

**Table 1:** Relative expression of known drug-resistance genes in doxorubicin resistant (DoxR) and vorinostat-treated doxorubicin-resistant (DoxR-v) cells compared to the parental lines.

		DoxR		DoxR-v	
Gene Symbol	Gene Entrez	SK-N-SH	SK-N-Be(2)C	SK-N-SH	SK-N-Be(2)C
ABCB1 (mdr1)	5243	4.63	4.23	1.98	2.04
ABCB6 (prp)	10058	1.68	1.81	1.57	1.81
ABCC3 (mrp3)	8714	N.D.	−2.39	N.D.	N.D.
ABCC4 (mrp4)	10257	2.17	2.12	N.D.	N.D.
ABCC5 (mrp5)	10057	−1.63	N.D.	−2.26	−2.68
ABCC9 (mrp9)	10060	−2.76	−4.15	−2.22	−2.89
BCL-2	596	1.53	N.D.	N.D.	N.D.
SIRT1	23411	1.75	2.02	N.D.	N.D.
BDNF	627	3.69	5.17	2.56	3.60
TH	7054	−1.87	−2.47	25.5	31.0

Results are expressed as a fold-change (all p<0.1). N.D. indicates no difference in gene expression (fold-change<1.5 and/or p>0.1). The following genes had no significant difference in any comparison: ABCC1 (MRP1), ABCC2 (MPR2), ABCC6 (mrp6), ABCC8 (mrp8), ABCC10 (mrp10), ABCC11 (mrp11), ABCC12 (mrp12), ABCC13 (mrp13), MGMT, SOD, HDAC1-8.

### Western Immunoblot

Whole cell lysis was performed by suspending cells in a lysis buffer (50 mmol/L HEPES (pH 7.4), 150 mmol/L NaCL, 100 mmol/L NaF, 1 mmol/L MgCl2, 1.5 mmol/L EGTA, 10% glycerol, 1% Triton X-100, 1 µg/ml leupeptin, 1 mm/l phenyl-methyl-sulfonyl-fluoride) and then removing insoluble material by centrifugation. Equal quantities of protein were separated by electrophoresis on 4–20% SDS-PAGE gels (Bio-rad; Hercules, CA) and transferred onto Immobilon-P membranes (Millipore, Bedford, MA). Proteins of interest were identified by reaction with specific primary antibodies for HIF-1α (BD Biosciences; Franklin Lakes, NJ), P-gp or beta-actin (Sigma-Aldrich, St. Louis, MO), followed by secondary antibodies linked to horseradish peroxidase (Promega, Madison, WI). Reactive bands were detected by chemiluminescence (Pierce/Thermo Fisher, Rockford, IL). When assessing for HIF-1α protein expression, HIF-1α protein was stabilized by treating cells with deferoxamine mesylate (DFO), 250 µM for 16 hours prior to whole cell lysis. Deferoxamine stabilizes HIF-1α by preventing normal oxygen-dependent proteosomal degradation.

**Figure 2:**
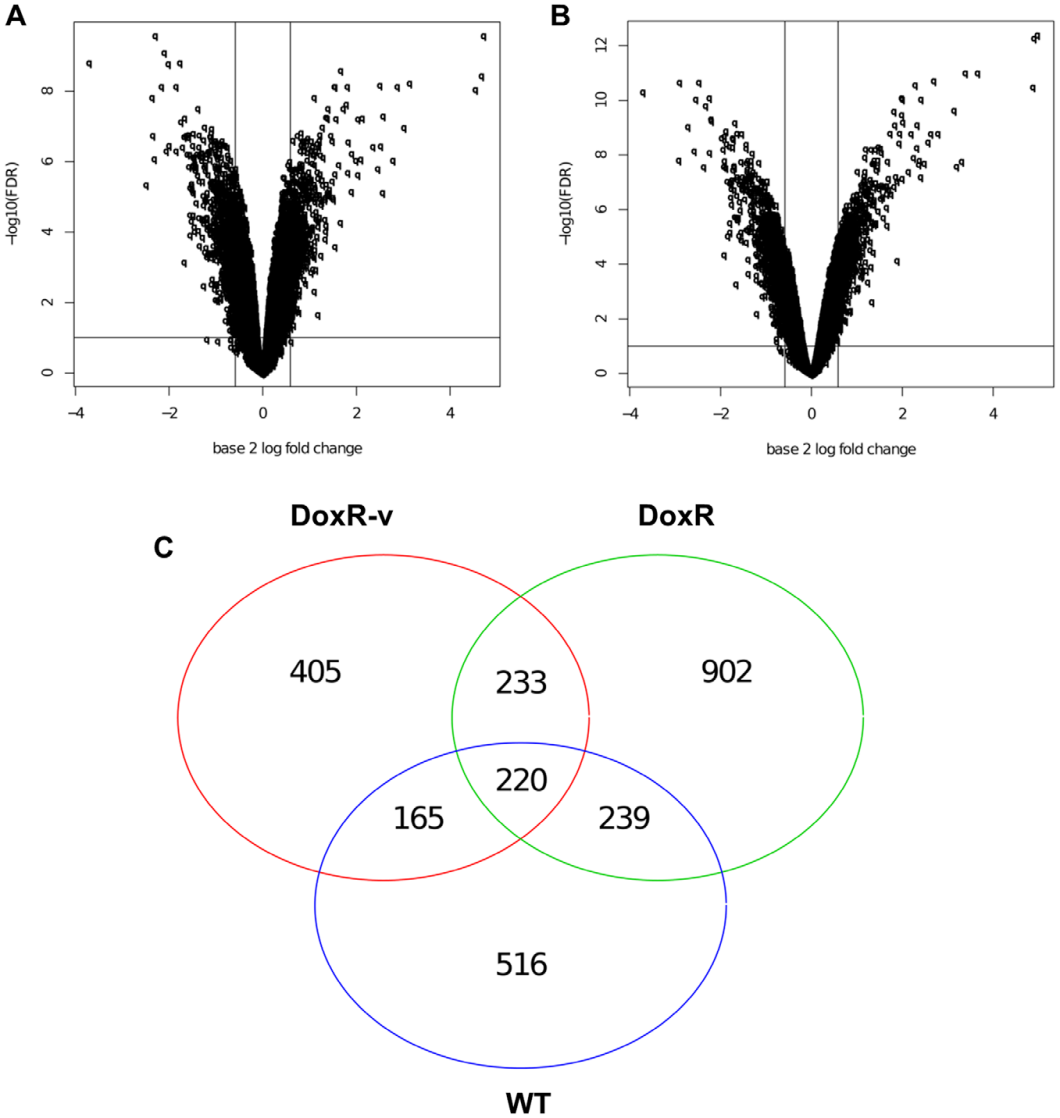
Distribution of differential expressed genes. Volcano plots illustrate the distribution of differentially expressed genes in the DoxR-v (a) SK-N-SH and (b) SK-N-Be(2)C cells relative to their parental (WT) lines. (c) Venn diagram shows the number of differentially expressed genes common to both cell lines for the DoxR-v, DoxR, and WT-v groups (all relative to their parental lines). There are 405 DEGs unique to the DoxR-v versus WT comparison for both cell lines.

**Table 2:** Genes with a potential role in doxorubicin-resistance following vorinostat treatment.

Gene Symbol	Gene Entrez ID	Gene Name	SK-N-SH Fold	SK-N-Be(2)C Fold
CSDA	8531	cold shock domain protein A	6.93	9.99
SST	6750	Somatostatin	5.94	9.24
PROK2	60675	prokineticin 2	3.57	4.61
PON2	5445	paraoxonase 2	3.40	3.94
DFNA5	1687	deafness, autosomal dominant 5	2.82	3.26
TUBB4	10382	tubulin, beta 4	2.76	3.35
C7orf58	79974	chromosome 7 open reading frame 58	2.57	3.55
FAM129B	64855	family with sequence similarity 129, member B	2.55	2.23
STOM	2040	Stomatin	2.44	2.40
LOC653888	653888	similar to Actin-related protein 2/3 complex subunit 1B (ARP2/3 complex 41 kDa subunit) (p41-ARC)	2.43	2.63
CORO2A	7464	coronin, actin binding protein, 2A	2.33	2.29
OSTF1	26578	osteoclast stimulating factor 1	2.26	2.30
SHISA5	51246	shisa homolog 5 (Xenopus laevis)	2.26	2.29
ASS1	445	argininosuccinate synthase 1	2.26	2.29
UCP2	7351	uncoupling protein 2 (mitochondrial, proton carrier)	2.21	2.33
SH2B3	10019	SH2B adaptor protein 3	2.18	2.21
CCNO	10309	cyclin O	2.16	2.63
C21orf63	59271	chromosome 21 open reading frame 63	2.16	2.78
CA10	56934	carbonic anhydrase X	2.16	2.31
MAP1LC3A	84557	microtubule-associated protein 1 light chain 3 alpha	2.12	2.43
EPAS1	2034	endothelial PAS domain protein 1	2.12	2.38
ENOSF1	55556	enolase superfamily member 1	2.12	2.47
HIBADH	11112	3-hydroxyisobutyrate dehydrogenase	2.10	2.36
ITM2A	9452	integral membrane protein 2A	2.06	2.59
RTTN	25914	Rotatin	2.04	2.25
SCARF2	91179	scavenger receptor class F, member 2	2.01	2.24
KIF1B	23095	kinesin family member 1B	2.00	2.10
CLIP3	25999	CAP-GLY domain containing linker protein 3	−2.00	−2.26
EBF3	253738	early B-cell factor 3	−2.02	−2.03
SNHG6	641638	small nucleolar RNA host gene 6 (non-protein coding)	−2.05	−2.15
LOC100131785	100131785	similar to ring finger protein 181	−2.11	−2.31
RNF181	51255	ring finger protein 181	−2.12	−2.24
FBXO8	26269	F-box protein 8	−2.12	−2.13
ST8SIA2	8128	ST8 alpha-N-acetyl-neuraminide alpha-2,8-sialyltransferase 2	−2.15	−2.69
ISOC1	51015	isochorismatase domain containing 1	−2.15	−2.18
SHD	56961	Src homology 2 domain containing transforming protein D	−2.15	−2.20
PAQR8	85315	progestin and adipoQ receptor family member VIII	−2.17	−2.26
GSTP1	2950	glutathione S-transferase pi 1	−2.25	−2.01
LOC644563	644563	general transcription factor IIIC, polypeptide 6, alpha 35 kDa pseudogene	−2.25	−2.13
SCARNA13	677768	small Cajal body-specific RNA 13	−2.33	−2.60
GTF3C6	112495	general transcription factor IIIC, polypeptide 6, alpha 35 kDa	−2.37	−2.56
ALG3	10195	asparagine-linked glycosylation 3, alpha-1,3- mannosyltransferasehomolog (S. cerevisiae)	−2.38	−2.51
EBPL	84650	emopamil binding protein-like	−2.38	−2.44
ATP6AP1	537	ATPase, H+ transporting, lysosomal accessory protein 1	−2.44	−2.58
NSDHL	50814	NAD(P) dependent steroid dehydrogenase-like	−2.45	−2.64
HMGCS1	3157	3-hydroxy-3-methylglutaryl-CoA synthase 1 (soluble)	−2.45	−2.80
RPF2	84154	ribosome production factor 2 homolog (S. cerevisiae)	−2.63	−2.63
SCARNA11	677780	small Cajal body-specific RNA 11	−2.73	−3.55
TNFRSF21	27242	tumor necrosis factor receptor superfamily, member 21	−2.84	−2.94
IL11RA	3590	interleukin 11 receptor, alpha	−3.25	−2.67
CALD1	800	caldesmon 1	−3.58	−4.60

There were 405 unique genes differentially expressed (fold change >1.5, adjusted p<0.1) in both SK-N-SH and SK-N-Be(2)C DoxR-v cells, but not in the DoxR or WT-v lines. The complete list of all 405 unique DEGs is available in Table S1, while the subset of those genes with the greatest differential expression (fold change>2) is listed here.

### Quantitative Real-time PCR

1 µg of RNA extracted from each cell lines as previous described was reverse transcribed to cDNA in a 20 µl reaction by using GeneAmp® RNA PCR Core Kit (Applied Biosystems, Foster City, CA) with random primers. Quantitative RT-PCR assays were then performed in triplicate on 1 µL of each cDNA dilution using the Power SYBR Green Master Mix (Applied Biosystems, Foster City, CA), the following thermal cycling specifications were performed on the ABI 7500 Fast Real-Time PCR system (Applied Biosystems, Foster City, CA) 60 s at 95°C and 40 cycles each for 9 s at 95°C and 60 s at 60°C. Specific intron-spanning primers were designed for EPAS1 (forward primer: CATTTGAGTCCTACCTGCTGC and reverse primer: GTAGAAGCCTGGCTCAGGTG), SST (forward primer: TTGTCCTCCCCACTTCTCT and reverse primer: AAAGATTTACTCAATAGAAACCA) and Pon2 (forward primer: AACAGCCATAGTAGTCAC and reverse primer: CAAGGGACAGAAAAGAAAG). The amplification of Glyceraldehyde-3-phosphate dehydrogenase (GAPDH) (forward primer: GGAGTGTGCCCGGGATGAAGAAT and reverse primer: TGGGGCTGGCAGGCTAAACA) was selected as the endogenous control to normalize all data, relative expression values were obtained by compared to the parental lines’ value.

**Figure 3:**
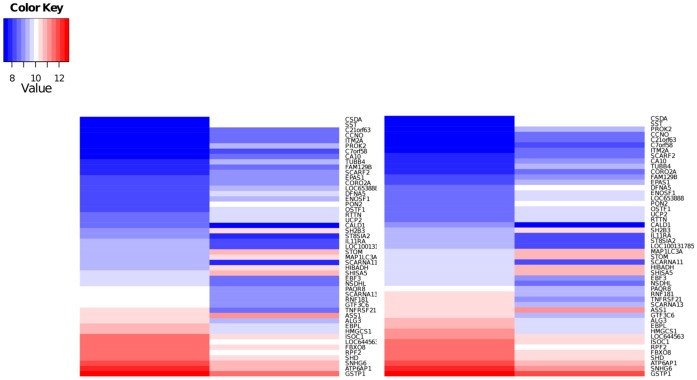
Relative expression of genes differentially expressed in doxorubicin-resistant compared to wild-type cells. Heat map for the SK-N-SH and SK-N-Be(2)C cell lines showing relative expression of the subset of the 405 DEGs unique to the DoxR-V versus WT comparison with a fold change >2.

**Figure 4:**
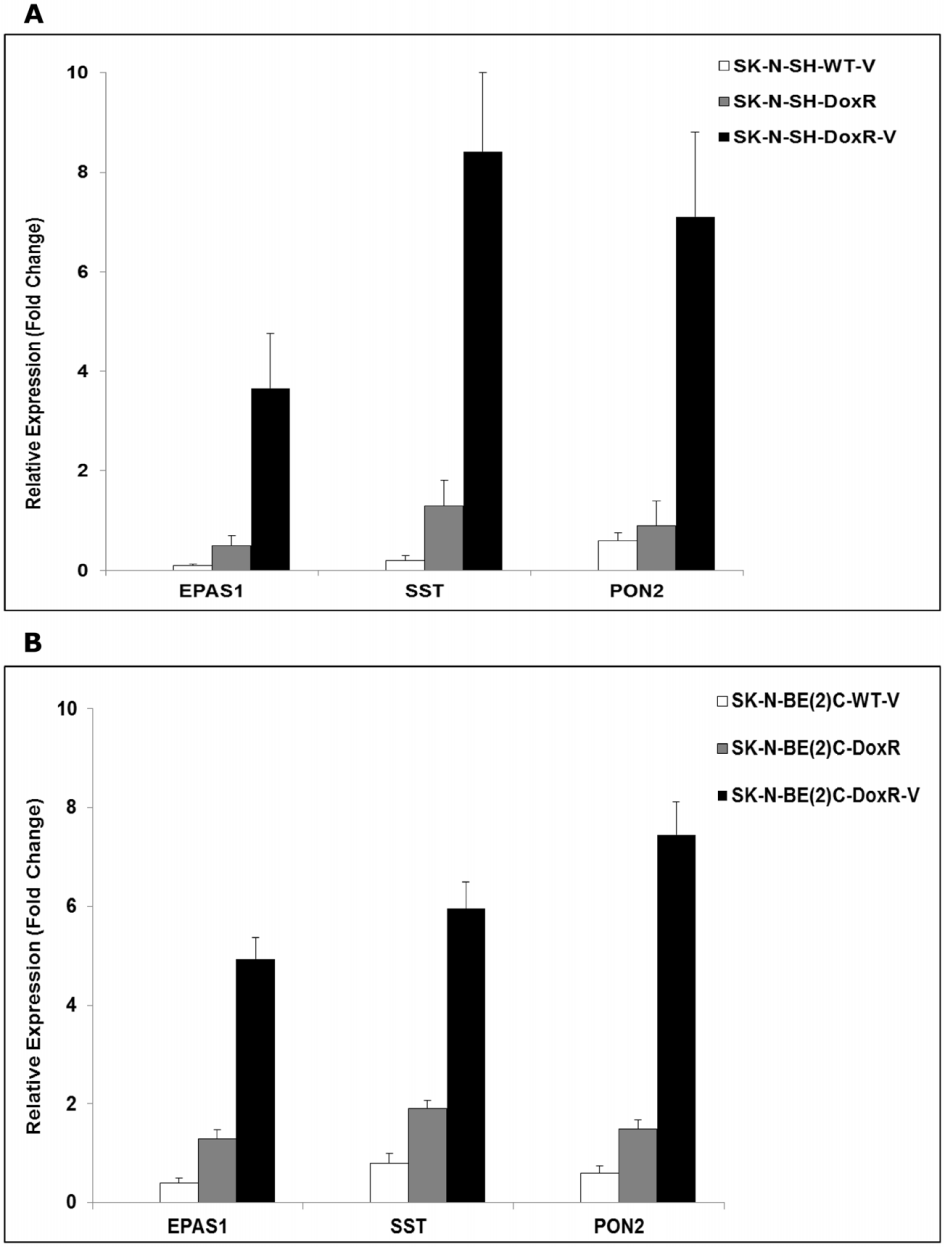
Expression of selected DRGs using qRT-PCR. Expression of endothelial PAS domain protein 1 (EPAS1), somatostatin (SST) and paraoxanase 2 (PON2) in (a) SK-N-SH and (b) SK-N-Be(2)C WT-v, DoxR and DoxR-v cells relative to their parental lines. Results were normalized to GAPDH by qRT-PCR and expressed as mean ± standard deviation for three biological repeats.

**Table 3:** Comparison of gene expression using microarray and qRT-PCR for selected DRGs.

	SK-N-SH Fold		SK-N-Be(2)C Fold	
Gene Name (Symbol)	Microarray	qRT-PCR	Microarray	qRT-PCR
Somatostatin (SST)	5.94	8.42	9.24	5.95
Paraoxanase 2 (PON2)	3.40	7.11	3.94	7.45
Endothelial PAS domain protein 1(EPAS1)	2.12	3.66	2.38	4.93

**Table 4:** Altered biological processes based on the genes with differential expression (fold change>1.5; adjusted p<0.1) unique to the DoxR-v cell lines as mapped by Gene Ontology.

Gene Ontology ID	Gene Ontology Biological Process	Annotated (n)	DEG (n)	Expected (n)	p
GO:0043648	dicarboxylic acid metabolic process	42	7	0.88	0.00002
GO:0044281	small molecule metabolic process	1698	57	35.58	0.0002
GO:0042278	purine nucleoside metabolic process	32	5	0.67	0.00049
GO:0046128	purine ribonucleoside metabolic process	32	5	0.67	0.00049
GO:0006732	coenzyme metabolic process	164	11	3.44	0.00068
GO:0008299	isoprenoid biosynthetic process	20	4	0.42	0.0007
GO:0042180	cellular ketone metabolic process	627	26	13.14	0.00073
GO:0051186	cofactor metabolic process	215	12	4.5	0.00196
GO:0006066	alcohol metabolic process	471	20	9.87	0.00225
GO:0031110	regulation of microtubule polymerization or depolymerization	27	4	0.57	0.00227
GO:0019752	carboxylic acid metabolic process	613	24	12.84	0.00249
GO:0043436	oxoacid metabolic process	613	24	12.84	0.00249
GO:0001101	response to acid	28	4	0.59	0.0026
GO:0006082	organic acid metabolic process	620	24	12.99	0.00288
GO:0009119	ribonucleoside metabolic process	50	5	1.05	0.00383
GO:0009215	purine deoxyribonucleoside triphosphate metabolic process	5	2	0.1	0.0042
GO:0021535	cell migration in hindbrain	5	2	0.1	0.0042
GO:0043570	maintenance of DNA repeat elements	5	2	0.1	0.0042
GO:0046827	positive regulation of protein export from nucleus	5	2	0.1	0.0042
GO:0007026	negative regulation of microtubule depolymerization	17	3	0.36	0.00498
GO:0031114	regulation of microtubule depolymerization	17	3	0.36	0.00498
GO:0031109	microtubule polymerization or depolymerization	35	4	0.73	0.00593
GO:0044262	cellular carbohydrate metabolic process	410	17	8.59	0.00594
GO:0006152	purine nucleoside catabolic process	6	2	0.13	0.00621
GO:0046130	purine ribonucleoside catabolic process	6	2	0.13	0.00621
GO:0033043	regulation of organelle organization	251	12	5.26	0.00677
GO:0006720	isoprenoid metabolic process	59	5	1.24	0.00777
GO:0031111	negative regulation of microtubule polymerization or depolymerization	20	3	0.42	0.00797
GO:0032465	regulation of cytokinesis	7	2	0.15	0.00857
GO:0042454	ribonucleoside catabolic process	7	2	0.15	0.00857
GO:0002027	regulation of heart rate	21	3	0.44	0.00916
GO:0007019	microtubule depolymerization	21	3	0.44	0.00916
GO:0009394	2'-deoxyribonucleotide metabolic process	21	3	0.44	0.00916
GO:0006084	acetyl-CoA metabolic process	40	4	0.84	0.00954

The number of genes annotated to each biological process category by Gene Ontology is shown in the third column. The number of actual differentially expressed genes (DEG) in each category is compared to the expected number of DEGs. P values were calculated by the Fischer exact test.

### Whole Genome Expression

RNA was isolated from triplicate specimens of each cell line using the RNeasy Mini Kit (Qiagen, Venlo, Netherlands). RNA quality and concentration were determined using the 2100 Bioanylzer (Agilent Technologies, Santa Clara, CA) and NanoDrop® ND-1000 spectrophotometer (Thermo Scientific, Wilmington, DE). Samples with RNA integrity numbers greater than 9.5 were used in subsequent expression analyses. All RNA samples were stored at −80°C prior to labeling and hybridization.

Amplification and labeling of the RNA was performed with the Illumina TotalPrep RNA Amplification Kit from Life Technologies (Ambion,Austin, TX) using 150 ng of RNA per sample. 750 ng of labeled cRNA were hybridized to Human HT-12 v4 Expression BeadChips for 18 hours and processed (Illumina, San Diego, CA) according to the manufacturer’s instructions. Slides were scanned using the Illumina iScan System Human HT-12 v4 beadchip containing 47,231 probes.

### Image and Data Analysis

Quality check and probe level processing of the Ilumina microarray data were further made with R bioconductor package, Lumi [11]. The data processing includes a normalization method [12] to reduce the obscuring variation between microarrays, which might be introduced during the processes of sample preparation, manufacturing, fluorescence labeling, hybridization and/or scanning. Hierarchical clustering and Principal Component Analysis were performed on the normalized signal data to assess the sample relationship and variability. Probes that were expressed in none of the samples as judged by the criteria of Illumina detection p-value ≥0.01 were filtered out. Differential gene expression between the different conditions was assessed by statistical linear model analysis using bioconductor package, limma, in which an empirical Bayes method is used to moderate the standard errors of the estimated log-fold changes of gene expression. It results in more stable inference and improved power, especially for experiments with small numbers of microarrays [13], [14]. The moderated t statistic p-values derived from the limma analysis above were further adjusted for multiple testing by Benjamini and Hochberg’s method to control false discovery rate (FDR). The list of differentially expressed genes was obtained by the FDR criteria of <10% and foldchange cutoff of >1.5, and visualized by volcanoplots. The gene ontology analysis of the lists of differentially expressed genes were performed with R bioconductor package, topGO [15]. All bioconductor packages are available at http://bioconductor.org and all computation was performed under R environment (http://www.r-project.org).

## Results

### Development of Drug Resistant Cells

The half maximal inhibitory concentration (IC_50_) of doxorubicin was determined by MTT assay. The IC_50_ of doxorubicin for the parental SK-N-SH and SK-N-Be(2)C cell lines was 3.6×10^−8^ M and 2.8×10^−7^ M, respectively. Drug-resistant (DoxR) cell lines were generated by incubating the parental cells with stepwise concentrations of doxorubicin, beginning 2 Log below the IC_50_. Cells were deemed resistant after surviving 5 passages in a concentration of drug approximately 2 Log above the IC_50_.

A parallel group of cells were treated with intermittent doses of vorinostat during the development of drug resistance. Cells were treated with their IC_25_ dose of vorinostat (1 µM for SK-N-SH and 0.5 µM for SK-N-Be(2)C) for 48 hours at the time of each logarithmic increase in the dose of doxorubicin. This dosing schedule was chosen to minimize the likelihood of simultaneously generating resistance to vorinostat. A control group of parental cells received only vorinostat at the same time as the treatment in the doxorubicin cells. Ultimately, four groups were generated: (1) parental or wild-type (WT), (2) vorinostat treated WT (WT-v), (3) doxorubicin resistant (DoxR), and (4) vorinostat-treated doxorubicin resistant (DoxR-v).

Resistance to doxorubicin was confirmed by MTT assay as shown in Figure 1 (a–b). The DoxR and DoxR-v cells were both equally resistant to doxorubicin, as well as cross-resistant to etoposide (Figure 1c) but not cisplatin (data not shown). Vorinostat treatment alone (WT-v cell lines) did not cause doxorubicin resistance.

P-glycoprotein (P-gp) expression was assessed by western blot (Figure 1d). P-gp, the protein product of the *ABCB1* or multidrug resistance *mdr1* gene was significantly upregulated in both the SK-N-SH and SK-N-Be(2)C DoxR cells relative to the WT or WT-V cells. Interestingly, although the DoxR-v cells were equally resistant to doxorubicin, these cells had reduced P-gp expression compared to their DoxR counterparts. Likewise in the SK-N-Be(2)C cell line, hypoxia inducible factor-1α (HIF-1α) expression was similarly upregulated in the Dox-R but not the DoxR-v cells (Figure 1e) under hypoxia mimicking conditions.

### Whole Genome Expression

Whole genome expression analysis was performed using the WT, WT-v, DoxR, and DoxR-v cells from the SK-N-SH and SK-N-Be(2)C lines. To understand the effect of vorinostat treatment on the development of drug resistance, we first analyzed the expression of known drug resistance genes in the DoxR and DoxR-v cells relative to their parental (WT) lines (Table 1). In agreement with the aforementioned western blot findings, expression of the *ABCB1* gene (which encodes the P-gp protein) was approximately 4-fold elevated in the DoxR cells, but only 2-fold elevated in the DoxR-v cells. Of note, expression of the *ABCC1* (mrp1) gene was not upregulated in the DoxR nor the DoxR-v cells relative to their parental line. No other known drug resistance genes had significant differential expression between the DoxR-v and DoxR cells to explain how the DoxR-v cells were equally resistant to doxorubicin despite having lower P-gp expression.

Genes differentially expressed in the DoxR-v cells relative to the parental (WT) lines were analyzed in an effort to determine alternative pathways for doxorubicin resistance following Vorinostat co-treatment. Volcano plots (Figure 2, a–b), demonstrate a similar pattern of differentially expressed genes (DEGs) in the SK-N-SH and SK-N-Be(2)C DoxR-v lines. DEGs common to both cell lines were compared between the DoxR-v, DoxR, and WT-v lines (Figure 2c). The 405 DEGs unique to the DoxR-v versus WT comparison were selected for further analysis, to hone in on the genetic changes responsible for drug resistance following this specific treatment combination. The 51 genes with the greatest differential expression (fold change >2) are shown in Table 2, and displayed visually as heat maps in Figure 3. The complete list of all 405 DEGs (fold change >1.5, p<0.1) unique to the DoxR-v versus WT comparison are shown in Table S1. Expression of these 405 DEGs in WT and DoxR-v cells for the SK-N-SH and SK-N-Be(2)C lines are displayed visually as heat maps in Figures S1 and S2, respectively. Among these DEGs, 181 (44.7%) were significantly down-regulated in the DoxR-v cells, while the remaining 224 (55.3%) were up-regulated.

To confirm the results of the whole genome analysis, qRT-PCR was performed on selected DRGs. Expression of somatostatin (SST), paraoxanase 2 (PON2) and endothelial PAS domain protein 1 (EPAS1) in the WT-v, DoxR and DoxR-v cells relative to their parental lines is shown in Figure 4. Relative expression of these genes based on microarray analysis and qRT-PCR is compared in Table 3.

### Functional Analysis

Functional analysis based on mapping of biological processes from the Gene Ontology Database was performed using the 405 DEGs unique to the DoxR-v versus WT comparison for both cell lines. For 34 different biological processes, a statistically greater than expected number of the genes annotated to that process were differentially expressed (Table 4). These altered biological processes included a predominance of those involved in cellular metabolism, cellular biosynthetic processes, and regulation of the cytoskeleton and microtubule structure. A hierarchical flowchart of these altered biological processes is included as Figure S3.

## Discussion

In the first study of the long-term effect of HDAC inhibition with vorinostat on the development of chemotherapy resistance, we herein demonstrate that vorinostat alters the mechanism but does not prevent the development of multidrug resistance. Cells treated intermittently with vorinostat each time the dose of doxorubicin was increased in generating resistance had significantly reduced expression of the *mdr1* gene and its protein product, P-gp, yet still demonstrated equivalent functional resistance to doxorubicin and etoposide. Whole genome analysis did not identify any known multidrug resistance genes that were upregulated in the vorinostat-treated cells to compensate for the lower expression of *mdr1.* However, several specific genes and biological processes that may play a role in the alternative mechanism of resistance following vorinostat treatment were identified.

Prior studies of histone deacetylase inhibitors, including Vorinostat, in neuroblastoma have focused on their short term effects where they have been shown to induce apoptosis of human neuroblastoma cells *in vitro* and *in vivo* [16], [17]. As vorinostat becomes more widely utilized in human clinical trials, it is important to understand the long-term effect of treatment with this drug on neuroblastoma responsiveness to standard cytotoxic agents [4], [10], [18]. Interest in the ability of HDAC inhibitors to prevent or reverse the development of drug resistance stems from findings of elevated HDAC expression in drug resistant neuroblastoma cell lines. Keshelava et al demonstrated that HDAC1 was upregulated in multidrug resistant neuroblastoma cell lines relative to drug-sensitive lines [19]. Inhibition of HDAC1 expression or activity resulted in synergistic killing of multidrug resistant cells when combined with standard cytotoxic agents. Oehme et al found elevated HDAC8 expression in primary human neuroblastoma samples from children with advanced and metastatic disease [20]. They reported that small-molecule inhibition of HDAC8 induced cellular differentiation and reduced proliferation. In both of the above studies, however, the effect of the HDAC inhibitors was studied in neuroblastoma cell lines that already displayed the multidrug resistance phenotype or other high-risk features. Our study is the first to investigate the effect of an HDAC inhibitor during induction of resistance to a commonly used cytotoxic agent by exposure to progressively increasing concentrations of the drug.

Our results provide further proof that expression of the P-gp drug efflux pump is only one component of the multi-drug resistance phenotype, which is also mediated by other drug efflux pumps and aspects of the cellular structure and tumor microenvironment [21]. We demonstrate that co-treatment with vorinostat can reduce expression of the *ABCB1 (mdr1)* gene and its protein product P-gp, yet these cells are equally resistant to doxorubicin and etoposide. We hypothesize that the effect of vorinostat on P-gp expression may be partially mediated by hypoxia inducible factor −1α (HIF-1α). We and others have demonstrated that histone deacetylase inhibitors, including vorinostat, can reduce HIF-1α activity in tumor cells [22], [23]. In turn, HIF-1α has been shown to bind to the promotor region of the *mdr1* gene to increase its expression under hypoxic conditions [24]. Therefore, long-term inhibition of HIF-1α activity by vorinostat treatment could partially explain the reduced expression of P-gp. At the same time, however, a recent prospective analysis of neuroblastoma tissue specimens suggested that the level of *mdr1* expression had no prognostic significance [6]. The present work confirms that neuroblastoma cells can develop equivalent multidrug resistance despite decreased P-gp expression.

We identified 405 additional genes with differential expression unique to the DoxR-v cells. Although no drug transporter proteins were included in this list, several of the identified genes have been previously shown to play a role in drug resistance. Endothelial PAS domain protein 1 (EPAS1), otherwise known as hypoxia-inducible factor 2α has been shown to play a key role in tumor responsiveness to chemotherapy. Elevation of HIF-2 (EPAS1) has been shown to suppress p53 function, and its knockdown can restore p53 function and reverse resistance to chemotherapy-induced cell death [25]. Furthermore, HIF-2 (EPAS1) has been shown to promote hypoxia response element (HRE)-driven gene expression, and shRNA knockdown increased renal cell carcinoma sensitivity to ionizing radiation [26]. If, as postulated above, HIF-1α activity is suppressed by vorinostat, which in turn leads to decreased P-gp expression, then increased HIF-2α (EPAS1) activity may mediate an alternative pathway by which the cells respond to the stress of the chemotherapeutic agents. Prior studies have shown that HIF-1α and HIF-2α activities have a reciprocal relationship: knockdown of HIF-1α induces HIF-2α (EPAS1) upregulation [27]. It is also known that HIF-2 activity is stimulated by Sirtuin 1 (sirt1), a class III HDAC that was upregulated in both our DoxR and DoxR-v cells, and which is not suppressed by vorinostat [28]. Further work is required to identify downstream targets of HIF-2α (EPAS1) which may mediate drug-resistance in vorinostat-treated cells.

A number of additional DEGs identified by our whole genome analysis have been previously postulated to play a role in chemotherapy resistance and other aspects of an aggressive tumor phenotype. Paraoxanose-2 upregulation has been found to correlate with chemotherapy resistance via an antioxidative mechanism which reduces oxidative damage to the endoplasmic reticulum [29]. Stomatin is a membrane protein that may serve as a skeleton anchor and regulate cation transport through lipid membranes, and it has been found upregulated in doxorubicin resistant tumors [30]. Finally, EBF3 has been identified as a tumor suppressor gene which mediates cell cycle arrest and apoptosis and whose expression is down regulated in a number of malignancies [31].

The genes with unique differential expression in the vorinostat co-treated doxorubicin-resistant cells are known to play key roles in a number of biological processes which have been implicated in the drug resistance phenotype. Of particular note, the DoxR-v cells were found to have statistically significant differential expression of genes involved in cellular metabolism, including ketone metabolism (GO:0042180) and lipid metabolism (GO:0006629). Alterations in the metabolic needs of tumor cells are increasingly recognized as a mechanism by which cells survive the stress of chemotherapy. Metabolic alterations in lipid metabolism have been proposed as an advantageous mechanism which helps tumor cells escape chemotherapy-induced apoptosis [32]. In drug-resistant colorectal cancer cells, alterations in lipid metabolism have been demonstrated and are postulated to offer a growth advantage under stress conditions [33]. Alterations in lipid metabolism may also alter drug uptake via changes in the properties of the cell’s lipid bilayer [21].

In conclusion, we have shown that intermittent therapy with Vorinostat has long-term effects on the development of resistance to doxorubicin in neuroblastoma cell lines. Vorinostat reduces expression of P-glycoprotein, the protein product of the *mdr1* gene, but does not prevent the functional development of multidrug resistance. Whole genome analysis identified several genes, including HIF-2α (EPAS1), whose differential expression might partially explain the equivalent multidrug resistance in the vorinostat treated cells. Further work is needed to investigate these differentially expressed genes, their downstream targets, and the biological process involved to fully delineate the implications of vorinostat on the long-term development of multidrug resistance in neuroblastoma.

## Supporting Information

Figure S1 **Heat map for the SK-N-SH cell line.** The heat map demonstrates relative expression of all 405 DEGs unique to the DoxR-V versus WT comparison.
(TIF)

Figure S2 **Heat map for the SK-N-Be(2)C cell line.** The heat map demonstrates relative expression of all 405 DEGs unique to the DoxR-V versus WT comparison.
(TIF)

Figure S3 **Flowchart of the biological processes involved by the 405 DEGs unique to the DoxR-v versus WT comparison**. Respective lines in each box display (1) the Gene Ontology ID, (2) The Gene Ontology biological process name, (3) the adjusted p value calculated by the Fisher exact test, and (4) the number of DEGs annotated to this process/the expected number of DEGs for this process.
(TIF)

Table S1 **List of all 405 unique genes differentially expressed (fold change >1.5, adjusted p<0.1) in both DoxR-v cell lines relative to their parental (WT) lines, and not differentially expressed in the DoxR or WT-v lines.**
(DOCX)
