# Supplementary figures and images for: The Effect of Vorinostat on the Development of Resistance to Doxorubicin in Neuroblastoma
Source: PLoS One. 2012 Jul 19;7(7):e40816. doi: 10.1371/journal.pone.0040816 (PMC3400660; doi:10.1371/journal.pone.0040816)

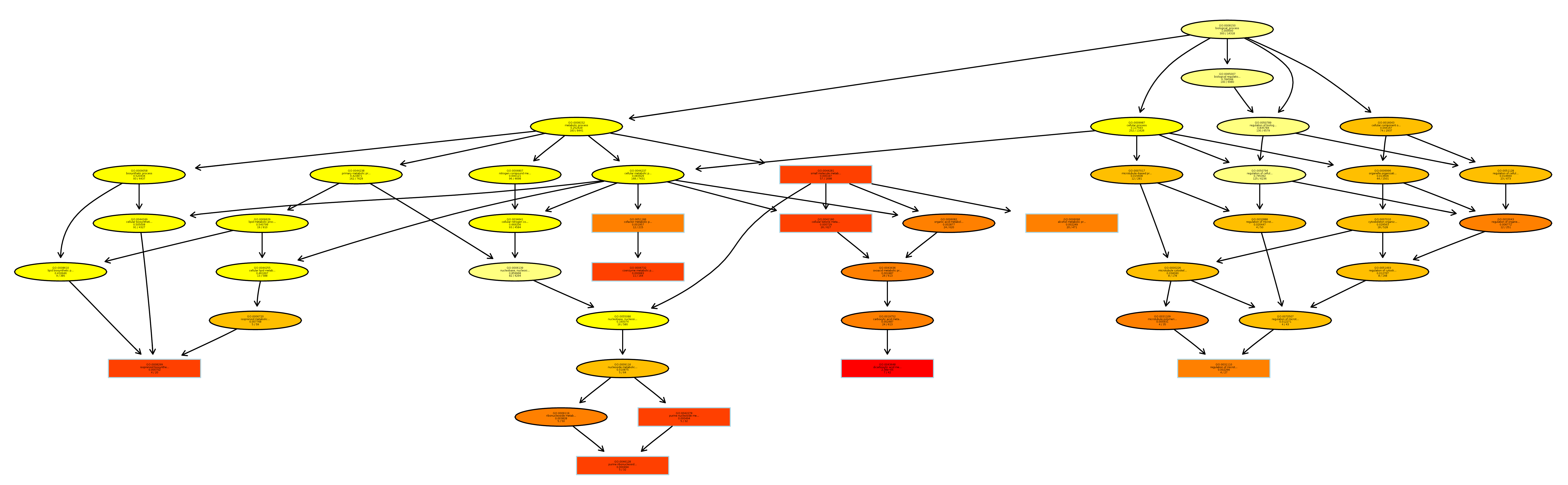

Supplement: Figure S3 — Flowchart of the biological processes involved by the 405 DEGs unique to the DoxR-v versus WT comparison. Respective lines in each box display (1) the Gene Ontology ID, (2) The Gene Ontology biological process name, (3) the adjusted p value calculated by the Fisher exact test, and (4) the number of DEGs annotated to this process/the expected number of DEGs for this process. (TIF) [file pone.0040816.s003.tif]
