# Supplementary material for: The Effect of Vorinostat on the Development of Resistance to Doxorubicin in Neuroblastoma
Source: PLoS One. 2012 Jul 19;7(7):e40816. doi: 10.1371/journal.pone.0040816 (PMC3400660; doi:10.1371/journal.pone.0040816)
Supplement: Table S1 — List of all 405 unique genes differentially expressed (fold change >1.5, adjusted p<0.1) in both DoxR-v cell lines relative to their parental (WT) lines, and not differentially expressed in the DoxR or WT-v lines. (DOCX) [file pone.0040816.s004.docx]

| **Gene Symbol** | **Gene Entrez ID** | **Gene Name** | **SK-N-SH Fold** | **SK-N-Be(2)C Fold** |
| --- | --- | --- | --- | --- |
| ABHD3 | 171586 | abhydrolase domain containing 3 | 1.75 | 1.88 |
| ACAD11 | 84129 | acyl-CoA dehydrogenase family, member 11 | 1.78 | 1.82 |
| ACADM | 34 | acyl-CoA dehydrogenase, C-4 to C-12 straight chain | 1.60 | 1.61 |
| ACN9 | 57001 | ACN9 homolog (S. cerevisiae) | 1.77 | 1.89 |
| ACOT4 | 122970 | acyl-CoA thioesterase 4 | 1.75 | 2.26 |
| ACSS2 | 55902 | acyl-CoA synthetase short-chain family member 2 | 1.81 | 1.89 |
| ACTL6B | 51412 | actin-like 6B | 1.69 | 1.81 |
| ADA | 100 | adenosine deaminase | 1.88 | 2.19 |
| ADRM1 | 11047 | adhesion regulating molecule 1 | 1.63 | 1.85 |
| AFAP1 | 60312 | actin filament associated protein 1 | -1.77 | -1.87 |
| AHCY | 191 | adenosylhomocysteinase | 1.60 | 1.64 |
| AHSA1 | 10598 | AHA1, activator of heat shock 90kDa protein ATPase homolog 1 (yeast) | 1.65 | 1.67 |
| AK3 | 50808 | adenylate kinase 3 | 1.61 | 1.66 |
| AKR1B1 | 231 | aldo-keto reductase family 1, member B1 (aldose reductase) | 1.77 | 1.74 |
| ALG3 | 10195 | asparagine-linked glycosylation 3, alpha-1,3- mannosyltransferase homolog (S. cerevisiae) | -2.38 | -2.51 |
| AMD1 | 262 | adenosylmethionine decarboxylase 1 | -1.77 | -1.69 |
| ANAPC11 | 51529 | anaphase promoting complex subunit 11 | -1.67 | -1.77 |
| ANGPTL2 | 23452 | angiopoietin-like 2 | -1.57 | -1.75 |
| ANKRD43 | 134548 | ankyrin repeat domain 43 | 1.90 | 1.92 |
| ARAP3 | 64411 | ArfGAP with RhoGAP domain, ankyrin repeat and PH domain 3 | -2.25 | -1.97 |
| ARHGDIA | 396 | Rho GDP dissociation inhibitor (GDI) alpha | 1.54 | 1.64 |
| ARL8B | 55207 | ADP-ribosylation factor-like 8B | 1.59 | 1.57 |
| ARMC10 | 83787 | armadillo repeat containing 10 | 1.78 | 1.93 |
| ARMCX1 | 51309 | armadillo repeat containing, X-linked 1 | -1.52 | -1.72 |
| ARPC1A | 10552 | actin related protein 2/3 complex, subunit 1A, 41kDa | 1.56 | 1.59 |
| ARPC1B | 10095 | actin related protein 2/3 complex, subunit 1B, 41kDa | 1.94 | 1.81 |
| ASS1 | 445 | argininosuccinate synthase 1 | 2.26 | 2.29 |
| ATG16L1 | 55054 | ATG16 autophagy related 16-like 1 (S. cerevisiae) | 1.54 | 1.61 |
| ATL3 | 25923 | atlastin GTPase 3 | 1.55 | 1.64 |
| ATP6AP1 | 537 | ATPase, H+ transporting, lysosomal accessory protein 1 | -2.44 | -2.58 |
| AVEN | 57099 | apoptosis, caspase activation inhibitor | 1.57 | 1.85 |
| B3GNT1 | 11041 | UDP-GlcNAc:betaGal beta-1,3-N-acetylglucosaminyltransferase 1 | -1.51 | -1.57 |
| B9D2 | 80776 | B9 protein domain 2 | -1.53 | -1.57 |
| BAI3 | 577 | brain-specific angiogenesis inhibitor 3 | -1.53 | -1.58 |
| BCAP29 | 55973 | B-cell receptor-associated protein 29 | 1.63 | 1.66 |
| BCHE | 590 | butyrylcholinesterase | -1.64 | -1.54 |
| BRD2 | 6046 | bromodomain containing 2 | -1.61 | -1.69 |
| BRI3 | 25798 | brain protein I3 | 1.52 | 1.68 |
| BRP44L | 51660 | brain protein 44-like | -1.72 | -1.60 |
| BTBD1 | 53339 | BTB (POZ) domain containing 1 | 1.63 | 1.73 |
| BTBD6 | 90135 | BTB (POZ) domain containing 6 | 1.67 | 1.67 |
| BUB1 | 699 | budding uninhibited by benzimidazoles 1 homolog (yeast) | 1.52 | 1.55 |
| C11orf17 | 56672 | chromosome 11 open reading frame 17 | 1.54 | 1.53 |
| C11orf67 | 28971 | chromosome 11 open reading frame 67 | -1.54 | -1.59 |
| C11orf70 | 85016 | chromosome 11 open reading frame 70 | -1.52 | -1.67 |
| C14orf142 | 84520 | chromosome 14 open reading frame 142 | 1.88 | 2.01 |
| C16orf48 | 84080 | chromosome 16 open reading frame 48 | -1.50 | -1.60 |
| C17orf95 | 124512 | chromosome 17 open reading frame 95 | 1.57 | 1.69 |
| C18orf56 | 494514 | chromosome 18 open reading frame 56 | 1.55 | 1.59 |
| C19orf10 | 56005 | chromosome 19 open reading frame 10 | -1.60 | -1.64 |
| C19orf12 | 83636 | chromosome 19 open reading frame 12 | -1.57 | -1.71 |
| C19orf60 | 55049 | chromosome 19 open reading frame 60 | -1.59 | -1.62 |
| C1orf61 | 10485 | chromosome 1 open reading frame 61 | 1.97 | 2.34 |
| C20orf103 | 24141 | chromosome 20 open reading frame 103 | -1.81 | -2.00 |
| C20orf108 | 116151 | chromosome 20 open reading frame 108 | 1.99 | 2.37 |
| C20orf201 | 198437 | chromosome 20 open reading frame 201 | 1.63 | 2.04 |
| C21orf63 | 59271 | chromosome 21 open reading frame 63 | 2.16 | 2.78 |
| C3orf70 | 285382 | chromosome 3 open reading frame 70 | 1.59 | 1.73 |
| C6orf48 | 50854 | chromosome 6 open reading frame 48 | -1.85 | -1.88 |
| C7orf47 | 221908 | chromosome 7 open reading frame 47 | 1.73 | 1.68 |
| C7orf58 | 79974 | chromosome 7 open reading frame 58 | 2.57 | 3.55 |
| CA10 | 56934 | carbonic anhydrase X | 2.16 | 2.31 |
| CALD1 | 800 | caldesmon 1 | -3.58 | -4.60 |
| CALM1 | 801 | calmodulin 1 (phosphorylase kinase, delta) | 1.52 | 1.65 |
| CBX4 | 8535 | chromobox homolog 4 | 1.57 | 1.66 |
| CCDC25 | 55246 | coiled-coil domain containing 25 | -1.57 | -1.68 |
| CCDC88C | 440193 | coiled-coil domain containing 88C | 1.82 | 2.63 |
| CCNO | 10309 | cyclin O | 2.16 | 2.63 |
| CDC16 | 8881 | cell division cycle 16 homolog (S. cerevisiae) | -1.69 | -1.85 |
| CDC40 | 51362 | cell division cycle 40 homolog (S. cerevisiae) | -1.67 | -1.86 |
| CDH11 | 1009 | cadherin 11, type 2, OB-cadherin (osteoblast) | 1.58 | 1.71 |
| CENPV | 201161 | centromere protein V | -1.87 | -2.05 |
| CETN2 | 1069 | centrin, EF-hand protein, 2 | -1.79 | -1.96 |
| CHAC2 | 494143 | ChaC, cation transport regulator homolog 2 (E. coli) | 1.51 | 1.80 |
| CHCHD8 | 51287 | coiled-coil-helix-coiled-coil-helix domain containing 8 | -1.53 | -1.65 |
| CKLF | 51192 | chemokine-like factor | 1.76 | 1.71 |
| CKMT1A | 548596 | creatine kinase, mitochondrial 1A | 1.80 | 1.57 |
| CKMT1B | 1159 | creatine kinase, mitochondrial 1B | 1.69 | 1.63 |
| CLIP3 | 25999 | CAP-GLY domain containing linker protein 3 | -2.00 | -2.26 |
| CMTM4 | 146223 | CKLF-like MARVEL transmembrane domain containing 4 | -1.54 | -1.64 |
| COPS4 | 51138 | COP9 constitutive photomorphogenic homolog subunit 4 (Arabidopsis) | -1.51 | -1.52 |
| COQ2 | 27235 | coenzyme Q2 homolog, prenyltransferase (yeast) | -1.54 | -1.72 |
| CORO2A | 7464 | coronin, actin binding protein, 2A | 2.33 | 2.29 |
| COTL1 | 23406 | coactosin-like 1 (Dictyostelium) | 1.75 | 2.62 |
| CPNE8 | 144402 | copine VIII | 1.75 | 1.78 |
| CSDA | 8531 | cold shock domain protein A | 6.93 | 9.99 |
| CSE1L | 1434 | CSE1 chromosome segregation 1-like (yeast) | 1.56 | 1.56 |
| CTDSP2 | 10106 | CTD (carboxy-terminal domain, RNA polymerase II, polypeptide A) small phosphatase 2 | -1.66 | -1.65 |
| CTNNA2 | 1496 | catenin (cadherin-associated protein), alpha 2 | -1.62 | -1.77 |
| CTTN | 2017 | cortactin | -1.63 | -1.78 |
| CYFIP2 | 26999 | cytoplasmic FMR1 interacting protein 2 | -1.69 | -2.52 |
| DDX24 | 57062 | DEAD (Asp-Glu-Ala-Asp) box polypeptide 24 | 1.60 | 1.65 |
| DFNA5 | 1687 | deafness, autosomal dominant 5 | 2.82 | 3.26 |
| DGUOK | 1716 | deoxyguanosine kinase | -1.79 | -1.96 |
| DHRS11 | 79154 | dehydrogenase/reductase (SDR family) member 11 | 1.51 | 1.64 |
| DHTKD1 | 55526 | dehydrogenase E1 and transketolase domain containing 1 | 1.58 | 1.68 |
| DHX30 | 22907 | DEAH (Asp-Glu-Ala-His) box polypeptide 30 | 1.78 | 1.73 |
| DLD | 1738 | dihydrolipoamide dehydrogenase | 1.60 | 1.73 |
| DLEU1 | 10301 | deleted in lymphocytic leukemia 1 (non-protein coding) | -1.51 | -1.55 |
| DNMT3B | 1789 | DNA (cytosine-5-)-methyltransferase 3 beta | 1.50 | 1.56 |
| DONSON | 29980 | downstream neighbor of SON | 1.70 | 1.77 |
| DPYSL4 | 10570 | dihydropyrimidinase-like 4 | 1.89 | 2.07 |
| DTNA | 1837 | dystrobrevin, alpha | 1.69 | 2.01 |
| E2F2 | 1870 | E2F transcription factor 2 | 1.61 | 1.54 |
| EBF1 | 1879 | early B-cell factor 1 | 1.92 | 2.01 |
| EBF3 | 253738 | early B-cell factor 3 | -2.02 | -2.03 |
| EBPL | 84650 | emopamil binding protein-like | -2.38 | -2.44 |
| EIF2C2 | 27161 | eukaryotic translation initiation factor 2C, 2 | -1.64 | -2.01 |
| EIF4E3 | 317649 | eukaryotic translation initiation factor 4E family member 3 | 1.61 | 1.68 |
| ELOVL6 | 79071 | ELOVL family member 6, elongation of long chain fatty acids (FEN1/Elo2, SUR4/Elo3-like, yeast) | -1.71 | -1.63 |
| EMD | 2010 | emerin | -1.61 | -1.64 |
| EML1 | 2009 | echinoderm microtubule associated protein like 1 | -1.57 | -1.73 |
| ENDOG | 2021 | endonuclease G | 1.77 | 1.92 |
| ENO2 | 2026 | enolase 2 (gamma, neuronal) | 1.87 | 1.62 |
| ENOSF1 | 55556 | enolase superfamily member 1 | 2.12 | 2.47 |
| EPAS1 | 2034 | endothelial PAS domain protein 1 | 2.12 | 2.38 |
| ETS1 | 2113 | v-ets erythroblastosis virus E26 oncogene homolog 1 (avian) | -1.57 | -1.92 |
| ETV5 | 2119 | ets variant 5 | -1.66 | -1.82 |
| EYA4 | 2070 | eyes absent homolog 4 (Drosophila) | -1.54 | -1.90 |
| FAM120AOS | 158293 | family with sequence similarity 120A opposite strand | 1.57 | 1.69 |
| FAM129B | 64855 | family with sequence similarity 129, member B | 2.55 | 2.23 |
| FAM167A | 83648 | family with sequence similarity 167, member A | -1.67 | -1.78 |
| FAM184A | 79632 | family with sequence similarity 184, member A | 1.58 | 1.73 |
| FAM50A | 9130 | family with sequence similarity 50, member A | -1.83 | -1.91 |
| FAM53C | 51307 | family with sequence similarity 53, member C | -1.54 | -1.51 |
| FAM58A | 92002 | family with sequence similarity 58, member A | -1.57 | -1.62 |
| FAM83D | 81610 | family with sequence similarity 83, member D | 1.51 | 1.53 |
| FAM96B | 51647 | family with sequence similarity 96, member B | -1.51 | -1.64 |
| FASTK | 10922 | Fas-activated serine/threonine kinase | 1.60 | 1.63 |
| FBXO8 | 26269 | F-box protein 8 | -2.12 | -2.13 |
| FHL2 | 2274 | four and a half LIM domains 2 | -1.54 | -1.55 |
| FIBP | 9158 | fibroblast growth factor (acidic) intracellular binding protein | -1.58 | -1.60 |
| FNBP1 | 23048 | formin binding protein 1 | -1.55 | -1.74 |
| FOXM1 | 2305 | forkhead box M1 | 1.51 | 1.69 |
| FOXO3 | 2309 | forkhead box O3 | -2.21 | -1.94 |
| FZD7 | 8324 | frizzled homolog 7 (Drosophila) | 1.71 | 1.71 |
| GABRG2 | 2566 | gamma-aminobutyric acid (GABA) A receptor, gamma 2 | 1.77 | 1.95 |
| GFRA2 | 2675 | GDNF family receptor alpha 2 | -1.54 | -1.58 |
| GLB1L2 | 89944 | galactosidase, beta 1-like 2 | 1.81 | 1.93 |
| GLIPR2 | 152007 | GLI pathogenesis-related 2 | -1.89 | -2.12 |
| GLS | 2744 | glutaminase | 1.59 | 1.65 |
| GNAI2 | 2771 | guanine nucleotide binding protein (G protein), alpha inhibiting activity polypeptide 2 | 1.87 | 1.94 |
| GNB2 | 2783 | guanine nucleotide binding protein (G protein), beta polypeptide 2 | 1.56 | 1.68 |
| GPR22 | 2845 | G protein-coupled receptor 22 | 1.54 | 1.60 |
| GPRC5C | 55890 | G protein-coupled receptor, family C, group 5, member C | 1.69 | 2.14 |
| GSS | 2937 | glutathione synthetase | 1.71 | 1.76 |
| GSTP1 | 2950 | glutathione S-transferase pi 1 | -2.25 | -2.01 |
| GTF3C6 | 112495 | general transcription factor IIIC, polypeptide 6, alpha 35kDa | -2.37 | -2.56 |
| H2AFJ | 55766 | H2A histone family, member J | -1.98 | -2.05 |
| HADH | 3033 | hydroxyacyl-CoA dehydrogenase | -1.65 | -1.66 |
| HAUS4 | 54930 | HAUS augmin-like complex, subunit 4 | 1.64 | 1.73 |
| HAUS7 | 55559 | HAUS augmin-like complex, subunit 7 | -1.62 | -1.74 |
| HDDC2 | 51020 | HD domain containing 2 | -1.62 | -1.56 |
| HIATL1 | 84641 | hippocampus abundant transcript-like 1 | 1.59 | 1.54 |
| HIBADH | 11112 | 3-hydroxyisobutyrate dehydrogenase | 2.10 | 2.36 |
| HIBCH | 26275 | 3-hydroxyisobutyryl-CoA hydrolase | 1.62 | 1.51 |
| HIGD1A | 25994 | HIG1 hypoxia inducible domain family, member 1A | 1.70 | 1.74 |
| HMGCR | 3156 | 3-hydroxy-3-methylglutaryl-CoA reductase | -1.65 | -1.51 |
| HMGCS1 | 3157 | 3-hydroxy-3-methylglutaryl-CoA synthase 1 (soluble) | -2.45 | -2.80 |
| HNRNPA1 | 3178 | heterogeneous nuclear ribonucleoprotein A1 | -1.54 | -1.67 |
| HOXC4 | 3221 | homeobox C4 | 1.66 | 1.70 |
| HS3ST2 | 9956 | heparan sulfate (glucosamine) 3-O-sulfotransferase 2 | 1.68 | 1.90 |
| HSPA1B | 3304 | heat shock 70kDa protein 1B | 1.79 | 1.87 |
| IDH3G | 3421 | isocitrate dehydrogenase 3 (NAD+) gamma | -1.90 | -1.92 |
| IFNGR1 | 3459 | interferon gamma receptor 1 | -1.57 | -1.61 |
| IL11RA | 3590 | interleukin 11 receptor, alpha | -3.25 | -2.67 |
| IL27RA | 9466 | interleukin 27 receptor, alpha | 1.71 | 1.80 |
| ING3 | 54556 | inhibitor of growth family, member 3 | 1.59 | 1.59 |
| INSIG1 | 3638 | insulin induced gene 1 | -1.51 | -1.70 |
| INTS12 | 57117 | integrator complex subunit 12 | -1.68 | -1.78 |
| ISOC1 | 51015 | isochorismatase domain containing 1 | -2.15 | -2.18 |
| ITGA6 | 3655 | integrin, alpha 6 | 1.55 | 1.90 |
| ITM2A | 9452 | integral membrane protein 2A | 2.06 | 2.59 |
| ITPA | 3704 | inosine triphosphatase (nucleoside triphosphate pyrophosphatase) | -1.58 | -1.51 |
| ITPK1 | 3705 | inositol 1,3,4-triphosphate 5/6 kinase | 1.72 | 1.92 |
| ITPKA | 3706 | inositol 1,4,5-trisphosphate 3-kinase A | 1.88 | 2.02 |
| ITPR3 | 3710 | inositol 1,4,5-triphosphate receptor, type 3 | 1.53 | 1.79 |
| JAM2 | 58494 | junctional adhesion molecule 2 | 1.54 | 1.66 |
| JMJD8 | 339123 | jumonji domain containing 8 | -1.61 | -1.70 |
| KCNMA1 | 3778 | potassium large conductance calcium-activated channel, subfamily M, alpha member 1 | 1.78 | 2.27 |
| KIAA0182 | 23199 | KIAA0182 | 1.54 | 1.60 |
| KIAA0391 | 9692 | KIAA0391 | 1.52 | 1.58 |
| KIAA1274 | 27143 | KIAA1274 | 1.57 | 2.00 |
| KIF1B | 23095 | kinesin family member 1B | 2.00 | 2.10 |
| KIF26A | 26153 | kinesin family member 26A | 1.51 | 1.79 |
| KLC1 | 3831 | kinesin light chain 1 | 1.97 | 1.94 |
| KTN1 | 3895 | kinectin 1 (kinesin receptor) | 1.86 | 1.92 |
| LAMA5 | 3911 | laminin, alpha 5 | 1.54 | 1.53 |
| LCLAT1 | 253558 | lysocardiolipin acyltransferase 1 | 1.67 | 1.61 |
| LINGO2 | 158038 | leucine rich repeat and Ig domain containing 2 | 1.92 | 2.31 |
| LMBR1 | 64327 | limb region 1 homolog (mouse) | 1.91 | 1.95 |
| LOC100131785 | 1E+08 | similar to ring finger protein 181 | -2.11 | -2.31 |
| LOC100133008 | 1E+08 | hypothetical LOC100133008 | -1.64 | -1.96 |
| LOC387820 | 387820 | similar to DnaJ (Hsp40) homolog, subfamily B, member 6 | 1.50 | 1.51 |
| LOC391578 | 391578 | MAF1 homolog (S. cerevisiae) pseudogene | -1.53 | -1.59 |
| LOC402175 | 402175 | hypothetical gene supported by AF044957; NM_004547 | -1.76 | -1.72 |
| LOC644563 | 644563 | general transcription factor IIIC, polypeptide 6, alpha 35kDa pseudogene | -2.25 | -2.13 |
| LOC645781 | 645781 | hypothetical protein LOC645781 | 1.52 | 1.67 |
| LOC653888 | 653888 | similar to Actin-related protein 2/3 complex subunit 1B (ARP2/3 complex 41 kDa subunit) (p41-ARC) | 2.43 | 2.63 |
| LOC728809 | 728809 | hypothetical LOC728809 | -1.76 | -1.75 |
| LRRC16A | 55604 | leucine rich repeat containing 16A | -1.50 | -1.86 |
| MAP1LC3A | 84557 | microtubule-associated protein 1 light chain 3 alpha | 2.12 | 2.43 |
| MAP7D1 | 55700 | MAP7 domain containing 1 | 1.62 | 1.71 |
| MAPT | 4137 | microtubule-associated protein tau | 1.94 | 2.02 |
| MCM10 | 55388 | minichromosome maintenance complex component 10 | 1.58 | 1.65 |
| ME2 | 4200 | malic enzyme 2, NAD(+)-dependent, mitochondrial | 1.58 | 1.59 |
| MED4 | 29079 | mediator complex subunit 4 | -1.68 | -1.71 |
| MFGE8 | 4240 | milk fat globule-EGF factor 8 protein | -1.59 | -1.81 |
| MID1 | 4281 | midline 1 (Opitz/BBB syndrome) | -1.66 | -2.14 |
| MMD | 23531 | monocyte to macrophage differentiation-associated | 1.72 | 1.78 |
| MOAP1 | 64112 | modulator of apoptosis 1 | 1.61 | 1.61 |
| MORF4L2 | 9643 | mortality factor 4 like 2 | -1.53 | -1.60 |
| MRPL47 | 57129 | mitochondrial ribosomal protein L47 | -1.75 | -1.76 |
| MRPS15 | 64960 | mitochondrial ribosomal protein S15 | 1.63 | 1.54 |
| MSH2 | 4436 | mutS homolog 2, colon cancer, nonpolyposis type 1 (E. coli) | 1.55 | 1.72 |
| MTHFD2 | 10797 | methylenetetrahydrofolate dehydrogenase (NADP+ dependent) 2, methenyltetrahydrofolate cyclohydrolase | -1.59 | -1.65 |
| MTPN | 136319 | myotrophin | 1.63 | 1.67 |
| MYH9 | 4627 | myosin, heavy chain 9, non-muscle | 1.69 | 1.84 |
| MYO19 | 80179 | myosin XIX | 1.93 | 1.97 |
| MZF1 | 7593 | myeloid zinc finger 1 | -1.55 | -1.77 |
| NBL1 | 4681 | neuroblastoma, suppression of tumorigenicity 1 | 1.79 | 2.01 |
| NCAM1 | 4684 | neural cell adhesion molecule 1 | -1.61 | -1.67 |
| NCAM2 | 4685 | neural cell adhesion molecule 2 | 1.84 | 1.98 |
| NCK2 | 8440 | NCK adaptor protein 2 | -1.67 | -1.77 |
| NCOR2 | 9612 | nuclear receptor corepressor 2 | 1.96 | 2.07 |
| NDRG4 | 65009 | NDRG family member 4 | -1.51 | -1.59 |
| NEK1 | 4750 | NIMA (never in mitosis gene a)-related kinase 1 | -1.55 | -1.58 |
| NELF | 26012 | nasal embryonic LHRH factor | -1.56 | -1.56 |
| NFIB | 4781 | nuclear factor I/B | -1.73 | -1.82 |
| NFKB1 | 4790 | nuclear factor of kappa light polypeptide gene enhancer in B-cells 1 | -1.57 | -1.65 |
| NIN | 51199 | ninein (GSK3B interacting protein) | 1.64 | 2.06 |
| NOMO2 | 283820 | NODAL modulator 2 | -1.51 | -1.57 |
| NPM3 | 10360 | nucleophosmin/nucleoplasmin 3 | -1.69 | -1.78 |
| NSDHL | 50814 | NAD(P) dependent steroid dehydrogenase-like | -2.45 | -2.64 |
| NT5DC2 | 64943 | 5'-nucleotidase domain containing 2 | 1.75 | 1.86 |
| NUBP1 | 4682 | nucleotide binding protein 1 (MinD homolog, E. coli) | -1.54 | -1.72 |
| NUDT14 | 256281 | nudix (nucleoside diphosphate linked moiety X)-type motif 14 | 1.63 | 1.68 |
| NUDT3 | 11165 | nudix (nucleoside diphosphate linked moiety X)-type motif 3 | -1.85 | -1.84 |
| OBFC2A | 64859 | oligonucleotide/oligosaccharide-binding fold containing 2A | 1.60 | 1.78 |
| OMA1 | 115209 | OMA1 homolog, zinc metallopeptidase (S. cerevisiae) | 1.56 | 1.75 |
| OSGEPL1 | 64172 | O-sialoglycoprotein endopeptidase-like 1 | 1.56 | 1.62 |
| OSTF1 | 26578 | osteoclast stimulating factor 1 | 2.26 | 2.30 |
| OTUB1 | 55611 | OTU domain, ubiquitin aldehyde binding 1 | 1.51 | 1.68 |
| PAQR8 | 85315 | progestin and adipoQ receptor family member VIII | -2.17 | -2.26 |
| PARP2 | 10038 | poly (ADP-ribose) polymerase 2 | 1.67 | 1.71 |
| PARVB | 29780 | parvin, beta | 1.74 | 1.85 |
| PCBP2 | 5094 | poly(rC) binding protein 2 | 1.56 | 1.92 |
| PCK2 | 5106 | phosphoenolpyruvate carboxykinase 2 (mitochondrial) | 1.72 | 1.78 |
| PDE4B | 5142 | phosphodiesterase 4B, cAMP-specific | 1.55 | 1.68 |
| PDE9A | 5152 | phosphodiesterase 9A | 1.61 | 1.68 |
| PDLIM3 | 27295 | PDZ and LIM domain 3 | -1.50 | -1.82 |
| PDSS2 | 57107 | prenyl (decaprenyl) diphosphate synthase, subunit 2 | -1.57 | -1.59 |
| PDXK | 8566 | pyridoxal (pyridoxine, vitamin B6) kinase | 1.53 | 1.57 |
| PFN1 | 5216 | profilin 1 | 1.57 | 1.66 |
| PGAM4 | 441531 | phosphoglycerate mutase family member 4 | -1.56 | -1.81 |
| PGRMC2 | 10424 | progesterone receptor membrane component 2 | -1.89 | -1.76 |
| PHLDA1 | 22822 | pleckstrin homology-like domain, family A, member 1 | 1.66 | 1.69 |
| PHLDB1 | 23187 | pleckstrin homology-like domain, family B, member 1 | -1.59 | -1.88 |
| PIAS4 | 51588 | protein inhibitor of activated STAT, 4 | -1.64 | -1.91 |
| PIP4K2A | 5305 | phosphatidylinositol-5-phosphate 4-kinase, type II, alpha | 1.69 | 1.72 |
| PJA2 | 9867 | praja ring finger 2 | 1.75 | 1.76 |
| PKN3 | 29941 | protein kinase N3 | 1.56 | 1.71 |
| PLD5 | 200150 | phospholipase D family, member 5 | 1.66 | 1.90 |
| PLEKHA6 | 22874 | pleckstrin homology domain containing, family A member 6 | -1.52 | -1.61 |
| PMPCB | 9512 | peptidase (mitochondrial processing) beta | 1.57 | 1.59 |
| POLE2 | 5427 | polymerase (DNA directed), epsilon 2 (p59 subunit) | 1.68 | 1.50 |
| POLR1C | 9533 | polymerase (RNA) I polypeptide C, 30kDa | -1.63 | -1.56 |
| POLR2H | 5437 | polymerase (RNA) II (DNA directed) polypeptide H | -1.90 | -1.92 |
| POLR3A | 11128 | polymerase (RNA) III (DNA directed) polypeptide A, 155kDa | 1.96 | 1.92 |
| PON2 | 5445 | paraoxonase 2 | 3.40 | 3.94 |
| POPDC3 | 64208 | popeye domain containing 3 | -1.84 | -2.00 |
| PPA2 | 27068 | pyrophosphatase (inorganic) 2 | -1.54 | -1.67 |
| PPDPF | 79144 | pancreatic progenitor cell differentiation and proliferation factor homolog (zebrafish) | 1.74 | 1.68 |
| PPP1R14A | 94274 | protein phosphatase 1, regulatory (inhibitor) subunit 14A | 1.54 | 1.87 |
| PRICKLE1 | 144165 | prickle homolog 1 (Drosophila) | 1.72 | 1.75 |
| PROK2 | 60675 | prokineticin 2 | 3.57 | 4.61 |
| PSMB10 | 5699 | proteasome (prosome, macropain) subunit, beta type, 10 | -1.59 | -1.66 |
| PSMD8 | 5714 | proteasome (prosome, macropain) 26S subunit, non-ATPase, 8 | -1.78 | -1.96 |
| PTGER2 | 5732 | prostaglandin E receptor 2 (subtype EP2), 53kDa | 1.71 | 1.73 |
| PTGR1 | 22949 | prostaglandin reductase 1 | 1.63 | 1.79 |
| PXMP2 | 5827 | peroxisomal membrane protein 2, 22kDa | 1.68 | 1.68 |
| RAB22A | 57403 | RAB22A, member RAS oncogene family | 1.81 | 1.69 |
| RAB35 | 11021 | RAB35, member RAS oncogene family | -1.50 | -1.60 |
| RAB5C | 5878 | RAB5C, member RAS oncogene family | 1.53 | 1.59 |
| RAMP2 | 10266 | receptor (G protein-coupled) activity modifying protein 2 | 1.81 | 2.19 |
| RAP1GDS1 | 5910 | RAP1, GTP-GDP dissociation stimulator 1 | -1.70 | -1.64 |
| RENBP | 5973 | renin binding protein | -1.61 | -1.80 |
| RFC2 | 5982 | replication factor C (activator 1) 2, 40kDa | 1.50 | 1.58 |
| RGAG4 | 340526 | retrotransposon gag domain containing 4 | -1.67 | -1.83 |
| RNF121 | 55298 | ring finger protein 121 | -1.61 | -1.67 |
| RNF181 | 51255 | ring finger protein 181 | -2.12 | -2.24 |
| ROD1 | 9991 | ROD1 regulator of differentiation 1 (S. pombe) | 1.74 | 1.82 |
| RPF2 | 84154 | ribosome production factor 2 homolog (S. cerevisiae) | -2.63 | -2.63 |
| RPL13A | 23521 | ribosomal protein L13a | -1.61 | -1.68 |
| RPL9 | 6133 | ribosomal protein L9 | -2.43 | -1.93 |
| RPS15A | 6210 | ribosomal protein S15a | -1.73 | -1.77 |
| RPS23 | 6228 | ribosomal protein S23 | -1.58 | -1.54 |
| RPS4X | 6191 | ribosomal protein S4, X-linked | -1.65 | -1.92 |
| RPS6KB2 | 6199 | ribosomal protein S6 kinase, 70kDa, polypeptide 2 | -1.59 | -1.51 |
| RTKN | 6242 | rhotekin | -1.50 | -1.70 |
| RTN1 | 6252 | reticulon 1 | 1.91 | 2.20 |
| RTTN | 25914 | rotatin | 2.04 | 2.25 |
| RXRB | 6257 | retinoid X receptor, beta | -1.75 | -1.91 |
| SCARF2 | 91179 | scavenger receptor class F, member 2 | 2.01 | 2.24 |
| SCARNA11 | 677780 | small Cajal body-specific RNA 11 | -2.73 | -3.55 |
| SCARNA13 | 677768 | small Cajal body-specific RNA 13 | -2.33 | -2.60 |
| SCPEP1 | 59342 | serine carboxypeptidase 1 | -1.55 | -1.59 |
| SDHA | 6389 | succinate dehydrogenase complex, subunit A, flavoprotein (Fp) | 1.66 | 1.63 |
| SDSL | 113675 | serine dehydratase-like | 1.67 | 2.11 |
| SEC23B | 10483 | Sec23 homolog B (S. cerevisiae) | 1.55 | 1.52 |
| SESN1 | 27244 | sestrin 1 | -1.56 | -1.79 |
| SH2B3 | 10019 | SH2B adaptor protein 3 | 2.18 | 2.21 |
| SHD | 56961 | Src homology 2 domain containing transforming protein D | -2.15 | -2.20 |
| SHISA5 | 51246 | shisa homolog 5 (Xenopus laevis) | 2.26 | 2.29 |
| SKA2 | 348235 | spindle and kinetochore associated complex subunit 2 | 1.52 | 1.61 |
| SLC25A13 | 10165 | solute carrier family 25, member 13 (citrin) | 1.79 | 1.76 |
| SLC2A1 | 6513 | solute carrier family 2 (facilitated glucose transporter), member 1 | -1.65 | -1.60 |
| SLC37A4 | 2542 | solute carrier family 37 (glucose-6-phosphate transporter), member 4 | -1.56 | -1.62 |
| SLC44A2 | 57153 | solute carrier family 44, member 2 | 1.60 | 1.93 |
| SMAGP | 57228 | small cell adhesion glycoprotein | 1.84 | 2.14 |
| SMNDC1 | 10285 | survival motor neuron domain containing 1 | 1.53 | 1.76 |
| SNAP25 | 6616 | synaptosomal-associated protein, 25kDa | 1.90 | 1.88 |
| SNHG6 | 641638 | small nucleolar RNA host gene 6 (non-protein coding) | -2.05 | -2.15 |
| SNORA64 | 26784 | small nucleolar RNA, H/ACA box 64 | -1.52 | -1.66 |
| SNORA76 | 677842 | small nucleolar RNA, H/ACA box 76 | -1.57 | -1.57 |
| SNORD104 | 692227 | small nucleolar RNA, C/D box 104 | -1.55 | -1.55 |
| SNORD80 | 26774 | small nucleolar RNA, C/D box 80 | 1.55 | 1.83 |
| SPOCK1 | 6695 | sparc/osteonectin, cwcv and kazal-like domains proteoglycan (testican) 1 | 1.79 | 1.96 |
| SSR4 | 6748 | signal sequence receptor, delta (translocon-associated protein delta) | -1.61 | -1.82 |
| SST | 6750 | somatostatin | 5.94 | 9.24 |
| ST6GALNAC4 | 27090 | ST6 (alpha-N-acetyl-neuraminyl-2,3-beta-galactosyl-1,3)-N-acetylgalactosaminide alpha-2,6-sialyltransferase 4 | 1.57 | 1.80 |
| ST6GALNAC5 | 81849 | ST6 (alpha-N-acetyl-neuraminyl-2,3-beta-galactosyl-1,3)-N-acetylgalactosaminide alpha-2,6-sialyltransferase 5 | 1.96 | 2.77 |
| ST8SIA2 | 8128 | ST8 alpha-N-acetyl-neuraminide alpha-2,8-sialyltransferase 2 | -2.15 | -2.69 |
| STAC2 | 342667 | SH3 and cysteine rich domain 2 | 1.67 | 2.09 |
| STC2 | 8614 | stanniocalcin 2 | -1.60 | -1.73 |
| STMN2 | 11075 | stathmin-like 2 | -1.62 | -1.75 |
| STOM | 2040 | stomatin | 2.44 | 2.40 |
| SUCLG1 | 8802 | succinate-CoA ligase, alpha subunit | -1.84 | -1.91 |
| SUGT1 | 10910 | SGT1, suppressor of G2 allele of SKP1 (S. cerevisiae) | -1.61 | -1.64 |
| SUV39H1 | 6839 | suppressor of variegation 3-9 homolog 1 (Drosophila) | -1.66 | -1.68 |
| SYT4 | 6860 | synaptotagmin IV | 2.09 | 1.94 |
| TANC1 | 85461 | tetratricopeptide repeat, ankyrin repeat and coiled-coil containing 1 | 1.52 | 1.61 |
| TAX1BP1 | 8887 | Tax1 (human T-cell leukemia virus type I) binding protein 1 | 1.61 | 1.85 |
| TCF7L2 | 6934 | transcription factor 7-like 2 (T-cell specific, HMG-box) | 1.66 | 1.93 |
| TERC | 7012 | telomerase RNA component | -1.61 | -2.03 |
| TFDP2 | 7029 | transcription factor Dp-2 (E2F dimerization partner 2) | -1.52 | -1.80 |
| TGFBR2 | 7048 | transforming growth factor, beta receptor II (70/80kDa) | 1.70 | 2.09 |
| TGOLN2 | 10618 | trans-golgi network protein 2 | -1.68 | -2.01 |
| TLE2 | 7089 | transducin-like enhancer of split 2 (E(sp1) homolog, Drosophila) | 1.92 | 2.37 |
| TLE4 | 7091 | transducin-like enhancer of split 4 (E(sp1) homolog, Drosophila) | 1.65 | 1.57 |
| TMED1 | 11018 | transmembrane emp24 protein transport domain containing 1 | -1.83 | -2.06 |
| TMED9 | 54732 | transmembrane emp24 protein transport domain containing 9 | -1.58 | -1.60 |
| TMEFF2 | 23671 | transmembrane protein with EGF-like and two follistatin-like domains 2 | 1.56 | 1.66 |
| TMEM100 | 55273 | transmembrane protein 100 | -1.53 | -1.93 |
| TMEM14A | 28978 | transmembrane protein 14A | -1.62 | -1.83 |
| TMEM151A | 256472 | transmembrane protein 151A | 1.81 | 1.90 |
| TMEM20 | 159371 | transmembrane protein 20 | 1.51 | 1.62 |
| TMEM204 | 79652 | transmembrane protein 204 | -1.63 | -1.91 |
| TMEM50B | 757 | transmembrane protein 50B | 1.64 | 1.64 |
| TMEM51 | 55092 | transmembrane protein 51 | -1.82 | -1.81 |
| TMOD2 | 29767 | tropomodulin 2 (neuronal) | -1.64 | -1.84 |
| TMUB1 | 83590 | transmembrane and ubiquitin-like domain containing 1 | 1.59 | 1.64 |
| TNFRSF21 | 27242 | tumor necrosis factor receptor superfamily, member 21 | -2.84 | -2.94 |
| TNRC6B | 23112 | trinucleotide repeat containing 6B | -1.50 | -1.62 |
| TPM1 | 7168 | tropomyosin 1 (alpha) | -1.77 | -1.93 |
| TPRKB | 51002 | TP53RK binding protein | -1.72 | -2.09 |
| TRIM13 | 10206 | tripartite motif-containing 13 | -1.53 | -1.71 |
| TRIM4 | 89122 | tripartite motif-containing 4 | 1.71 | 1.61 |
| TRO | 7216 | trophinin | -1.64 | -1.82 |
| TRPC4AP | 26133 | transient receptor potential cation channel, subfamily C, member 4 associated protein | 1.50 | 1.58 |
| TSC22D1 | 8848 | TSC22 domain family, member 1 | -1.86 | -1.85 |
| TSHZ1 | 10194 | teashirt zinc finger homeobox 1 | 1.65 | 1.67 |
| TTF2 | 8458 | transcription termination factor, RNA polymerase II | 1.55 | 1.55 |
| TUBB4 | 10382 | tubulin, beta 4 | 2.76 | 3.35 |
| TUFM | 7284 | Tu translation elongation factor, mitochondrial | 1.58 | 1.60 |
| UBAC2 | 337867 | UBA domain containing 2 | -1.83 | -1.91 |
| UBL4A | 8266 | ubiquitin-like 4A | -1.54 | -1.66 |
| UBR7 | 55148 | ubiquitin protein ligase E3 component n-recognin 7 (putative) | 1.70 | 1.72 |
| UCKL1 | 54963 | uridine-cytidine kinase 1-like 1 | 1.55 | 1.65 |
| UCP2 | 7351 | uncoupling protein 2 (mitochondrial, proton carrier) | 2.21 | 2.33 |
| ULK1 | 8408 | unc-51-like kinase 1 (C. elegans) | -1.66 | -1.76 |
| USO1 | 8615 | USO1 vesicle docking protein homolog (yeast) | -1.55 | -1.67 |
| UTP11L | 51118 | UTP11-like, U3 small nucleolar ribonucleoprotein, (yeast) | 1.60 | 1.64 |
| VGF | 7425 | VGF nerve growth factor inducible | 1.71 | 1.70 |
| VPS24 | 51652 | vacuolar protein sorting 24 homolog (S. cerevisiae) | -1.60 | -1.80 |
| VRK1 | 7443 | vaccinia related kinase 1 | 1.59 | 1.77 |
| WARS | 7453 | tryptophanyl-tRNA synthetase | 1.75 | 1.79 |
| WASL | 8976 | Wiskott-Aldrich syndrome-like | 1.83 | 1.93 |
| WBP2 | 23558 | WW domain binding protein 2 | 1.53 | 1.51 |
| WDR34 | 89891 | WD repeat domain 34 | 1.59 | 1.80 |
| WDR54 | 84058 | WD repeat domain 54 | -1.90 | -2.00 |
| WRB | 7485 | tryptophan rich basic protein | 1.78 | 1.76 |
| XRCC3 | 7517 | X-ray repair complementing defective repair in Chinese hamster cells 3 | 1.80 | 1.97 |
| YPEL3 | 83719 | yippee-like 3 (Drosophila) | -1.50 | -1.59 |
| YTHDF1 | 54915 | YTH domain family, member 1 | 1.65 | 1.53 |
| YWHAG | 7532 | tyrosine 3-monooxygenase/tryptophan 5-monooxygenase activation protein, gamma polypeptide | 1.51 | 1.55 |
| ZBTB33 | 10009 | zinc finger and BTB domain containing 33 | -1.56 | -1.56 |
| ZDHHC11 | 79844 | zinc finger, DHHC-type containing 11 | -1.52 | -1.69 |
| ZDHHC6 | 64429 | zinc finger, DHHC-type containing 6 | 1.53 | 1.58 |
| ZMYM6 | 9204 | zinc finger, MYM-type 6 | 1.80 | 1.80 |
| ZNF274 | 10782 | zinc finger protein 274 | -1.63 | -1.99 |
| ZNF280D | 54816 | zinc finger protein 280D | -1.51 | -1.68 |
| ZNF322A | 79692 | zinc finger protein 322A | -1.55 | -1.77 |
| ZNF330 | 27309 | zinc finger protein 330 | -1.91 | -1.60 |
| ZNF581 | 51545 | zinc finger protein 581 | -1.78 | -1.76 |
